# Supplementary material for: Scheduling mechanisms to control the spread of COVID-19
Source: PLoS One. 2022 Sep 15;17(9):e0272739. doi: 10.1371/journal.pone.0272739 (PMC9477360; doi:10.1371/journal.pone.0272739)
Supplement: S1 Appendix — (PDF) [file pone.0272739.s001.pdf]

A Appendix

A.1 Glossary of abbreviations

- T_p — Transmission probability, the probability that the disease transfers from a person to a neighbor in a round
- (g, d, t) — schedule
  - g — number of groups
  - d number of days a group is continuously scheduled
  - t — gap between schedules
- work ratio — ratio of average number of in-person working hours to the average number of in-person working hours pre-COVID (i.e., the $(1, 5, 2)$ schedule)
- $k(v)$ — degree of node v in contact graph
- C — mean number of contacts per person per day
- p — Chung-Lu parameter, the probability that the next neighbor is "close"
- $R(t)$ — reproductive rate, the average number of individuals an infected individual infects over the course of their infection
- D — average number of days a person remains infected

A.2 Formula for in-person work ratio

As defined in Item 2, the *in-person work* ratio or simply the *work* ratio is the *ratio* of the number of (in-person) working hours of an individual under our mechanism to the number of working hours under the standard five-day working week (i.e., the $(1, 5, 2)$ schedule), assuming that an individual works (in-person) for a fixed number of hours (say, 8) on each day they are working.

Theorem 2. *The work ratio for the (g, d, t) mechanism is $\frac{7}{5}\left(\frac{d}{gd+t}\right)$.*

Proof. Consider a group schedule (g, d, t). We note that a member of group i will wait $(g-1)d + t$ days quarantined after their final day unquarantined in a given cycle

$$\underbrace{0, 0, \ldots, 0}_{d \text{ days}}\underbrace{1, 1 \ldots, 1 \ldots g-1, g-1 \ldots g-1}_{(g-1)d \text{ days}}\underbrace{\emptyset\emptyset \ldots \emptyset}_{t \text{ days}}$$

In such a cycle, there are $gd + t$ days and each group works d days. Assuming that each day one works for a fixed number of hours, the fraction of the work hours to the total number of work hours possible is $\frac{d}{gd+t}$. For a standard $(1, 5, 2)$ schedule (5-day work week), the above ratio is $5/7$. Hence the work ratio for a (g, d, t) schedule is $\frac{7}{5}\left(\frac{d}{gd+t}\right)$. □

A.3 Relating reproductive rate to transmission probability

Let C be the (average) number of contacts per person (we assume this value to be 13.4 throughout this paper based on the data of Mossong et al. [1]) and D be the number of days a person remains infectious (we assume in this paper that this number is 11 days for COVID-19 [2]).

Contact graph model For the Contact Graph Model, one can approximately relate T_p and $R(t)$ as follows:

$$R(t) = (1 - (1 - T_p)^D) \times C$$

That is, since each individual u can infect a given neighbor v each day with probability T_p and hence not infect with probability $1 - T_p$, the probability that v is *not* infected after D days is $(1 - T_p)^D$. Thus, the probability that v is infected during the course of u's infection is $1 - (1 - T_p)^D$. Thus, on average, an individual u infects $(1 - (1 - T_p)^D) \times C$ individuals.

Random mixing model For the Random Mixing Model, one can approximately relate T_p and $R(t)$ as follows:

$$R(t) = T_p \times C \times D$$

That is, we assume that an individual has C new contacts on average per day on *each* of the D days. Since each contact has probability T_p of being infected, the formula follows.[6]

A.4 Omitted proofs of Section 2.3

Proof of Lemma 1

Proof. If an infected individual v is symptomatic, then

$$D^g = \max\{0, \min\{(d-2), 5\}\} + \max\{0, \sum_{i=1,2,\ldots} \min\{5 - (d + (g-1)d + t)i, d\}\}.$$

The first term $\min\{(d-2), 5\}$ comes from the fact that the group (which goes out first) is not infectious for the first two days and that $d - 2$ cannot be more than 5 since a symptomatic patient can be put in quarantine on the 5th day (see Section 2.2). Also it cannot be negative, so we take the maximum between 0 and $\min\{(d-2), 5\}$. The second term shows the repeated process of all the groups going out for work. The term $5 - (d + (g-1)d + t) = 5 - (gd + t)$ comes from the schedule equation (Theorem 2) and the fact that a symptomatic patient can be put in quarantine on the 5th day. Also it cannot be negative and hence the maximum function.

Also it is easy to see that $C^g = C/g = 13.4/g$, since we assume $C = 13.4$. Thus we get from Eq. (1):

$$\begin{aligned}
\mu_s &= T_p \cdot D^g \cdot C^g \\
&= T_p \cdot \left(d' + \sum_i d'_i\right) \cdot \frac{13.4}{g} \\
&= \frac{(13.4)T_p}{g} \cdot \left(d' + \sum_i d'_i\right)
\end{aligned}$$

□

Proof of Lemma 2

Proof. If v is asymptomatic, then D^g can be calculated as follows:

$$D^g = \max\{0, \min\{d-2, 11\}\} + \max\{0, \sum_{i=1,2,\ldots} \min\{11 - (d + (g-1)d + t)i, d\}\}.$$

The first term $\min\{d-2, 11\}$ comes from the fact that the group (which goes out first) is not infectious for the first two days and that $d-2$ cannot be more than 11 as we assume that a person can not be infectious for more than 11 days (see Section 2.2). The second term shows the repeated process of all the groups going out for work. The term $11-(d+(g-1)d+t) = 11-(gd+t)$ comes from the equation (Theorem 2) and the fact that a person can not be infectious for more than 11 days. Both the terms cannot be negative; so the maximum function is applied before them.

C^g is fixed, i.e., $C^g = C/g = 13.4/g$. Thus we get from Eq. (1):

$$\begin{aligned}
\mu_a &= T_p \cdot D^g \cdot C^g \\
&= T_p \left(d'' + \sum_i d''_i \right) \frac{13.4}{g} \\
&= \frac{(13.4)T_p}{g} \left(d'' + \sum_i d''_i \right)
\end{aligned}$$

References

1. Mossong J, Hens N, Jit M, Beutels P, Auranen K, Mikolajczyk R, et al. Social Contacts and Mixing Patterns Relevant to the Spread of Infectious Diseases. PLOS Medicine. 2008;5(3):1–1.
2. Ferguson N, Laydon D, Nedjati Gilani G, Imai N, Ainslie K, Baguelin M, et al. Impact of non-pharmaceutical interventions to reduce COVID-19 mortality and healthcare demand. Imperial College London. 2020;.
