# Supplementary material for: Scheduling mechanisms to control the spread of COVID-19
Source: PLoS One. 2022 Sep 15;17(9):e0272739. doi: 10.1371/journal.pone.0272739 (PMC9477360; doi:10.1371/journal.pone.0272739)
Supplement: Attachment — Submitted filename: Review-PONE-D-21-33787.pdf [file pone.0272739.s002.pdf]

Referee's report on article # PONE-D-21-33787
Scheduling Mechanisms to Control Spread of Covid-19

In this paper, the authors studied scheduling mechanisms that explore the trade-off between containing the spread of COVID12 19 and performing in-person activity in organizations. The proposed mechanisms are based on partitioning the population randomly 14 into groups and scheduling each group on appropriate days with possible gaps. Finally, the authors demonstrated the efficacy of our mechanisms by theoretical analysis and 24 extensive experimental simulations on various epidemiological models based on real-world data.

The results involved in the work are new and through which several special cases can be concluded; i.e. the approach and techniques can be modified in a straightforward manner to study the spread of other diseases, including COVID-19 variants.

Furthermore, they analyzed a number of group scheduling mechanisms that showcase the trade off between in-person work ratio and the disease spread.

Finally, I strongly recommend the publication of this paper in PLOS ONE.
