# Supplementary material for: Scheduling mechanisms to control the spread of COVID-19
Source: PLoS One. 2022 Sep 15;17(9):e0272739. doi: 10.1371/journal.pone.0272739 (PMC9477360; doi:10.1371/journal.pone.0272739)
Supplement: Attachment — Submitted filename: Response to Reviewers.pdf [file pone.0272739.s003.pdf]

Below is the original review, with our responses directly below relevant sections in red.

PONE-D-21-33787 Scheduling Mechanisms to Control Spread of Covid-19 PLOS ONE

Journal Requirements:

When submitting your revision, we need you to address these additional requirements.

1. Please ensure that your manuscript meets PLOS ONE's style requirements, including those for file naming. The PLOS ONE style templates can be found at https://journals.plos.org/plosone/s/file?id=wjVg/PLOSOne_formatting_sample_main_body.pdf and https://journals.plos.org/plosone/s/file?id=ba62/PLOSOne_formatting_sample_title_authors_affiliations.pdf

The manuscript and file names have been updated to follow the style requirements of PLOS ONE.

2. We note that the grant information you provided in the 'Funding Information' and 'Financial Disclosure' sections do not match.

When you resubmit, please ensure that you provide the correct grant numbers for the awards you received for your study in the 'Funding Information' section.

The funding information provided in the submission is correct.

3. Thank you for stating the following in the Acknowledgments Section of your manuscript:

"John Augustine's research is supported in part by an Extra-Mural Research Grant (file number EMR/2016/003016) funded by the Science and Engineering Research Board, Department of Science and Technology, Government of India and by the VAJRA faculty program of the Government of India. Anisur Rahaman Molla's research supported by DST Inspire Faculty research grant DST/INSPIRE/04/2015/002801. Gopal Pandurangan's research is supported, in part, by NSF grants IIS-1633720, CCF-BSF-1717075, CCF1540512, US-Israel BSF award 2016419, and by the VAJRA faculty program of the Government of India."

We note that you have provided funding information that is not currently declared in your Funding Statement. However, funding information should not appear in the Acknowledgments section or other areas of your manuscript. We will only publish funding information present in the Funding Statement section of the online submission form. Please remove any funding-related text from the manuscript and let us know how you would like to update your Funding Statement. Currently, your Funding Statement reads as follows:

"John Augustine's research is supported in part by an Extra-Mural Research Grant (file number

EMR/2016/003016) funded by the Science and Engineering Research Board, Department of Science and Technology, Government of India and by the VAJRA faculty program of the Government of India.

Anisur Rahaman Molla's research supported by DST Inspire Faculty research grant DST/INSPIRE/04/2015/002801.

Gopal Pandurangan's research is supported, in part, by NSF grants IIS-1633720, CCF-BSF-1717075, CCF-1540512, US-Israel BSF award 2016419, and by the VAJRA faculty program of the Government of India.

https://nsf.gov/ https://www.bsf.org.il/ https://www.vajra-india.in/

The funders had no role in study design, data collection and analysis, decision to publish, or preparation of the manuscript."

Please include your amended statements within your cover letter; we will change the online submission form on your behalf.

We have removed the funding information from the acknowledgments section. Additionally, the funding information above is correct. We restate it below:

John Augustine's research is supported in part by an Extra-Mural Research Grant (file number EMR/2016/003016) funded by the Science and Engineering Research Board, Department of Science and Technology, Government of India and by the VAJRA faculty program of the Government of India.

Anisur Rahaman Molla's research supported by DST Inspire Faculty research grant DST/INSPIRE/04/2015/002801.

Gopal Pandurangan's research is supported, in part, by NSF grants IIS-1633720, CCF-BSF-1717075, CCF-1540512, US-Israel BSF award 2016419, and by the VAJRA faculty program of the Government of India.

The funders had no role in study design, data collection and analysis, decision to publish, or preparation of the manuscript.

The funding information is also included in the cover letter.

4. Please review your reference list to ensure that it is complete and correct. If you have cited papers that have been retracted, please include the rationale for doing so in the manuscript text, or remove

these references and replace them with relevant current references. Any changes to the reference list should be mentioned in the rebuttal letter that accompanies your revised manuscript. If you need to cite a retracted article, indicate the article's retracted status in the References list and also include a citation and full reference for the retraction notice.

Additional Editor Comments:

To Corresponding Author, I require before a possible acceptance that are followed all the modifications suggested by the referees. So, I recommend you to read the reports and make the changes. There are no conflicts between the review. My decision of MINOR REVISION is justified on PLOS ONE journal according to the reports. Best regards

[Note: HTML markup is below. Please do not edit.]

Reviewers' comments:

Reviewer's Responses to Questions

Comments to the Author

We thank the reviewers very much for their insightful comments. We have addressed them and responses are below.

1. Is the manuscript technically sound, and do the data support the conclusions?

The manuscript must describe a technically sound piece of scientific research with data that supports the conclusions. Experiments must have been conducted rigorously, with appropriate controls, replication, and sample sizes. The conclusions must be drawn appropriately based on the data presented.

Reviewer #1: Yes

Reviewer #2: Yes

2. Has the statistical analysis been performed appropriately and rigorously?

Reviewer #1: Yes

Reviewer #2: Yes

3. Have the authors made all data underlying the findings in their manuscript fully available?

The PLOS Data policy requires authors to make all data underlying the findings described in their manuscript fully available without restriction, with rare exception (please refer to the Data Availability Statement in the manuscript PDF file). The data should be provided as part of the manuscript or its supporting information, or deposited to a public repository. For example, in addition to summary

statistics, the data points behind means, medians and variance measures should be available. If there are restrictions on publicly sharing datae.g. participant privacy or use of data from a third partythose must be specified.

Reviewer #1: Yes

Reviewer #2: Yes

4. Is the manuscript presented in an intelligible fashion and written in standard English?

PLOS ONE does not copyedit accepted manuscripts, so the language in submitted articles must be clear, correct, and unambiguous. Any typographical or grammatical errors should be corrected at revision, so please note any specific errors here.

Reviewer #1: Yes

Reviewer #2: Yes

5. Review Comments to the Author

Please use the space provided to explain your answers to the questions above. You may also include additional comments for the author, including concerns about dual publication, research ethics, or publication ethics. (Please upload your review as an attachment if it exceeds 20,000 characters)

Reviewer #1: In this paper, the authors studied scheduling mechanisms that explore the trade-off between containing the spread of COVID12 19 and performing in-person activity in organizations. The proposed mechanisms are based on partitioning the population randomly 14 into groups and scheduling each group on appropriate days with possible gaps. Finally, the authors demonstrated the efficacy of our mechanisms by theoretical analysis and 24 extensive experimental simulations on various epidemiological models based on real-world data. The results involved in the work are new and through which several special cases can be concluded; i.e. the approach and techniques can be modified in a straightforward manner to study the spread of other diseases, including COVID-19 variants. Furthermore, they analyzed a number of group scheduling mechanisms that showcase the trade off between in-person work ratio and the disease spread. Finally, I strongly recommend the publication of this paper in PLOS ONE.

We appreciate the reviewer's comments.

Reviewer #2: Nowadays, the work is very interesting. During winter the circulation of the virus is beginning to be higher with the consequences on health. The ongoing COVID-19 pandemic produced an unprecedented health and economic crisis, urging for the development of adapted actions aimed to

monitoring the spread of the new coronavirus. A new variant is spreading and the danger of a possible lock down have to be considered in the coming days. The proposed model of group scheduling has the aim to maintain the activity in presence and operating a control of the spread of the virus. This model is a useful idea for the rulers.

In the text it is well illustrated which are the advantages in group scheduling even if the groups are few and small. Monitoring the evolution with the time of reproductive rate constitutes a critical factor in situations such as that of COVID-19, when decisions need to be taken and action need to be made under emergency. Referring to the epidemiological model to predict disease spread by estimating Tp o Rt we can cite (A, B, C).

We thank the reviewer for suggesting these relevant citations and have added them to the manuscript.

In the real world, it may be useful to highlight the possible different effects of the group scheduling proposed to avoid contagion in different situations whereas the usual cyclical mechanisms typically do not. In the transmission of the virus must also be considered the possible influences determined by the behaviours more or less correct observed by the subjects. The authors have shown that Model 2.5.0 compared to Model 1.5.2, drastically reduces the number of peak infections and significantly the total number of infections in the population, but results in a 30% reduction in the work ratio. The proposed scheme would be able to achieve the result of reducing the transmission of the virus and reducing the contagion in health facilities or hospitals, even where the behaviours have not substantially changed a cause of pandemic (D).

We thank the reviewer for the insight regarding health facilities and hospitals and have updated the manuscript accordingly, as well as adding the suggested citation.

This is why the model could usefully be proposed in the construction of shifts of health workers. In the healthcare facilities, bearing the alternation provided for in the group scheduling mechanism presupposes a workforce that is numerically adequate to the standard needs. The latter could, with smaller health workers' numbers, in the event of a pandemic, cope with a greater activity with sacrifice but would allow to continue to safeguard health and provide essential services.

The identification of the three parameters for the identification of the group scheduling mechanism is clear. In my opinion, Table 1 does not add anything to what is described in the text. I would suggest to

create a Glossary of abbreviations containing, in addition to the parameters used in the analysis, also the other acronyms and abbreviations used in the text for easier reading (g,d,t, n, k, v, u, R, X, m...etc.)

We have added a glossary of abbreviations to the appendix.

Suggested references

A.Abry P, Pustelnik N, Roux S, Jensen P, Flandrin P, Gribonval R, Lucas CG, Guichard É, Borgnat P, Garnier N. Spatial and temporal regularization to estimate COVID-19 reproduction number R(t): Promoting piecewise smoothness via convex optimization. PLoS One. 2020 Aug 20;15(8):e0237901. doi: 10.1371/journal.pone.0237901.

B.Ponjavi M, Karabegovi A, Ferhatbegovi E, Tahirovi E, Uzunovi S, Travar M, Pilav A, Muli M, Karaka S, Avdi N, Mulabdi Z, Pavi G, Bio M, Vasilj I, Mami D, Huki M. Spatio-temporal data visualization for monitoring of control measures in the prevention of the spread of COVID-19 in Bosnia and Herzegovina. Med Glas (Zenica). 2020 Aug 1;17(2):265-274. doi: 10.17392/1215-20.

C.Hong HG, Li Y. Estimation of time-varying reproduction numbers underlying epidemiological processes: A new statistical tool for the COVID-19 pandemic. PLoS One. 2020 Jul 21;15(7):e0236464. doi: 10.1371/journal.pone.0236464.

D.Ragusa R, Marranzano M, Lombardo A, Quattrocchi R, Bellia MA, Lupo L. Has the COVID 19 Virus Changed Adherence to Hand Washing among Healthcare Workers? Behav Sci (Basel). 2021 Apr 15;11(4):53. doi: 10.3390/bs11040053.

We have added these references to the manuscript.

6. PLOS authors have the option to publish the peer review history of their article (what does this mean?). If published, this will include your full peer review and any attached files.

If you choose no, your identity will remain anonymous but your review may still be made public.

Do you want your identity to be public for this peer review? For information about this choice, including consent withdrawal, please see our Privacy Policy.

Reviewer #1: Yes: Mohamed I. Abbas

Reviewer #2: No

[NOTE: If reviewer comments were submitted as an attachment file, they will be attached to this email and accessible via the submission site. Please log into your account, locate the manuscript record, and check for the action link "View Attachments". If this link does not appear, there are no attachment

files.]

While revising your submission, please upload your figure files to the Preflight Analysis and Conversion Engine (PACE) digital diagnostic tool, https://pacev2.apexcovantage.com/. PACE helps ensure that figures meet PLOS requirements. To use PACE, you must first register as a user. Registration is free. Then, login and navigate to the UPLOAD tab, where you will find detailed instructions on how to use the tool. If you encounter any issues or have any questions when using PACE, please email PLOS at figures@plos.org. Please note that Supporting Information files do not need this step.
